# Supplementary material for: Linoleate-Enrichment of Mitochondrial Cardiolipin Molecular Species Is Developmentally Regulated and a Determinant of Metabolic Phenotype
Source: Biology (Basel). 2022 Dec 24;12(1):32. doi: 10.3390/biology12010032 (PMC9855531; doi:10.3390/biology12010032)
Supplement: Supplementary file 1 [file biology-12-00032-s001.zip › biology-2102889-supplementary.pdf]

Supplementary Materials:

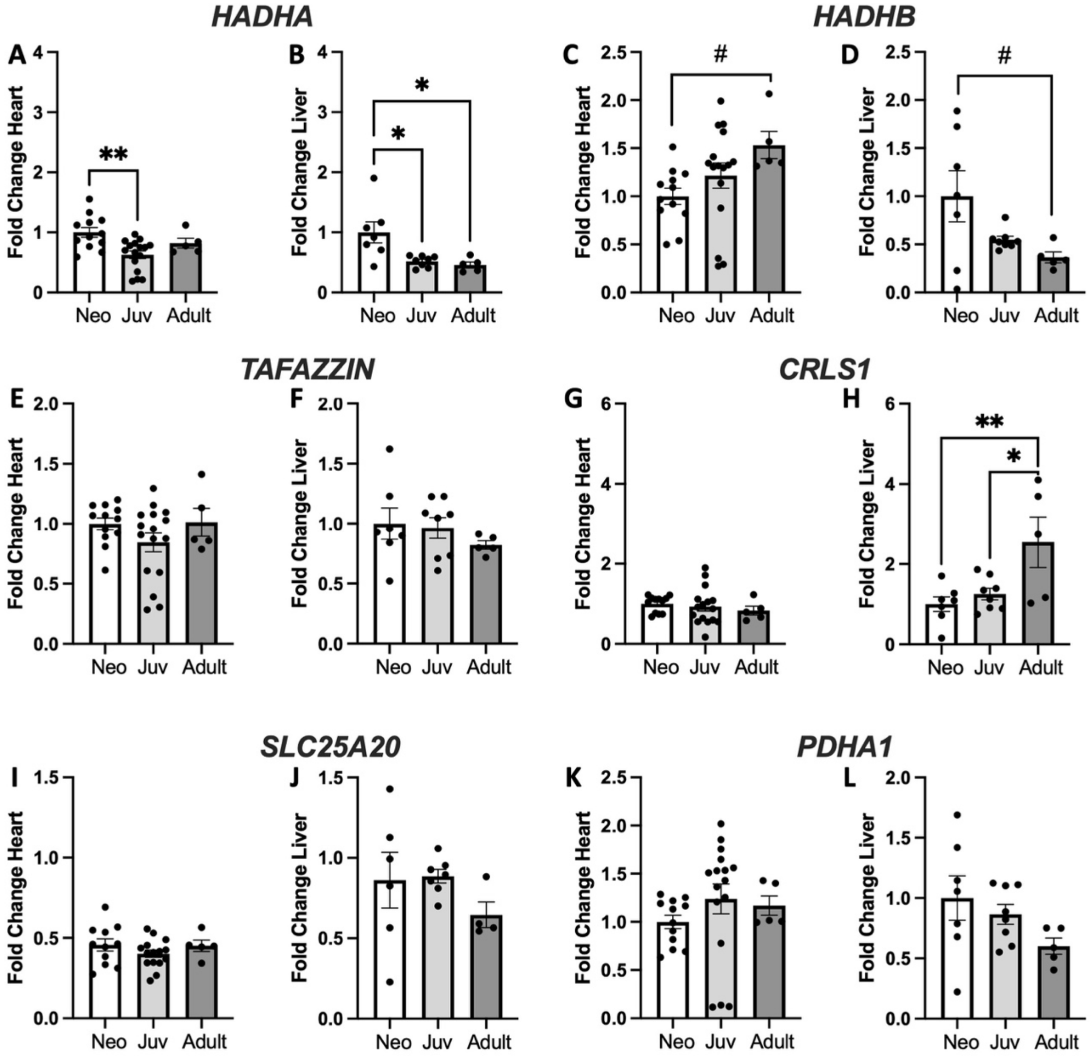

Figure S1. Additional genes in heart and liver during early development. Quantitative rtPCR for the heart and liver genes: HADHA, coding for Trifunctional Protein Alpha Subunit (A,B); HADHB, coding for Trifunctional Protein Beta Subunit (C,D); CL remodeling gene TAFFAZIN (E,F); CL biosynthesis gene CRLS1 (Cardiolipin Synthase) (G,H); SLC25A20 coding for Carnitine Acylcarnitine Translocase (I,J); PDHA1 coding for Pyruvate Dehydrogenase E1alpha subunit (K,L) #$p<0.1$, *$p<0.05$, **$p<0.01$; N=5-16 separate animals; Neo, neonatal; Juv, juvenile. Error bars represent standard error of the mean.

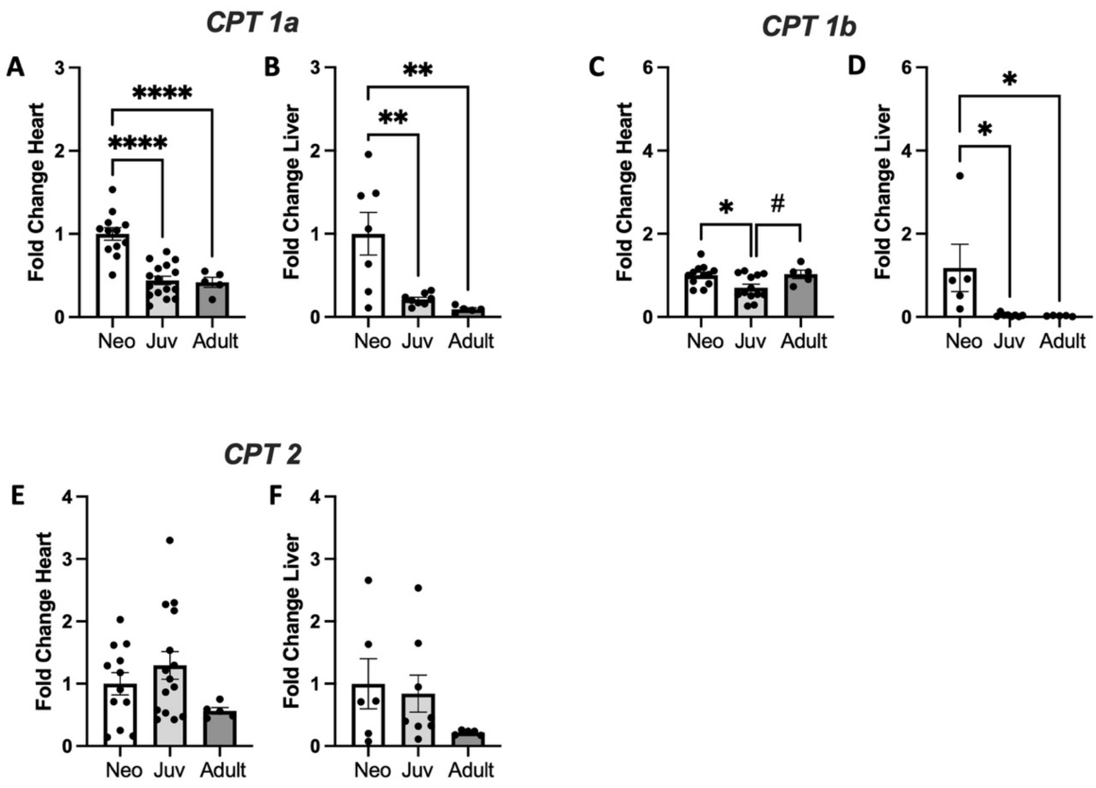

Figure S2. Carnitinepalmitoyltransferase (CPT) genes in heart and liver during early development. Quantitative rtPCR for the heart and liver genes: CPT1a, codes for the liver isoform of CPT I (A,B); CPT1b, codes for the muscle isoform of CPT I (C,D); CPT2, codes for CPT II (E,F). #p<0.1, *p<0.05, **p<0.01, ****p<0.0001; N=5-16 separate animals. Error bars represent standard error of the mean.

Table S1: Sequences Used for rt-qPCR Studies

Target	Accession Number	Forward (5' to 3')	Reverse (5' to 3')
CRLS1	NM_001014258.1	GTGGGAAGTGTAAAGGGATACA	GTAACTGAGAGGCTGGAACTAC
HADHA	NM_130826.2	CAAGGTTTGTGAATGAGGCAG	ACCATACAGATCCACAAAGCG
HADHB	NM_133618.3	CGCCTGTCCTTACTCACTAAA	CCATTCGAGAAACAGCAAAGG
TAFAZZIN	NM_001025748.1	GACTCCTAATTCCTGCTGGATAC	AGTGTAAGGGACAGCCAAAG
ACADL	NM_012819.2	GAAGTGATTCCCTACCACGAAG	CGCCATGTTTCTCTGCAATG
MCAD	NM_016986.2	CGCCCCAGACTACGATAAAAG	CACGCATCAAAAGTTCCCAG
PDHA1	NM_001004072.2	CTACAGACTTACCGCTACCATG	GCTGTTCACCATTCTATCCTTGAG
CD36	NM_031561.2	TACCTGTGAGTTGGCAAGAAG	CACCAATAACGGCTCCAGTAA
SLC27A1	NM_053580.2	GATGTGCTCTATGACTGCCTAC	CACCGTTAACCCGTAGATGATAC
SLC25A20	NM_053965.2	GTTGACTGAAGGCCCTACTTAC	CTGGGTTAGCTGGTTGAGAATAG
SLC2A1	NM_138827.2	CCCTGCAGTTCGGCTATAAC	GAGTGTGGTGAGTGTGGTG
CPT1a	NM_031559.2	CAGTGAGGACCTAAAGCAGAG	GGTGACGGTGAACTGGAAG
CPT1b	NM_013200.2	AGGCAGTAGCTTTCCAGTTC	CACACCCCTAAGGATACCATTC
CPT2	NM_012930.1	CATGCACTACCAGGACAGC	CCAACGCCAGTCTCAAAATTC
18s	NR_046237.2	GCCGCTAGAGGTGAAATTCTTG	CTTTCGCTCTGGTCCGTCTT
